# Using Virtual Reality (VR) Mock-Ups for Evidence-Based Healthcare Facility Design Decisions

**DOI:** 10.3390/ijerph182111250

**Published:** 2021-10-26

**Authors:** Jonas Shultz, Rajesh Jha

**Affiliations:** 1 Health Quality Council of Alberta, Calgary, AB T2N 2A4, Canada; 2 Department of Anesthesiology, Perioperative and Pain Management, Faculty of Medicine, Cumming School of Medicine, University of Calgary, Calgary, AB T2N 1N4, Canada; 3 SimInsights Inc., Irvine, CA 92618, USA; rkjha1@siminsights.com

**Keywords:** virtual reality, mock-up, healthcare facility design, human factors, simulation

## Abstract

(1) Background: There are many complexities and trade-offs that design teams consider when designing or renovating a built environment for healthcare. Virtual reality (VR) mock-ups can allow design teams to evaluate the planned design. This study aimed to examine the overall value of using VR mock-ups to conduct a simulation-based mock-up evaluation. (2) Methods: Data collected from scenario enactments within a VR mock-up was compared to data collected from an existing medication room with the same design to assess predictive validity. Outcomes regarding quality and patient safety were also examined as a result of design modifications to the VR mock-up which were identified through a post-occupancy evaluation (POE) of the existing medication room. Survey data from participants, hospital design stakeholders, and POE recommendation recipients captured perceptions regarding the evaluation process. Specifically, this included perceptions regarding mock-up and scenario realism as well as utility of the evaluation process. (3) Results: Evidence-based data collected using the VR mock-up accurately assessed workflow (link analysis), bumps, impediments, interruptions, and task completion times. Collecting data pertaining to selection errors and equipment placement were identified after procuring the VR software and therefore the accuracy of these measures was not assessed. Searching behaviours were not possible to capture using the VR software. A 506% return on investment was achieved through the VR mock-up evaluations. (4) Conclusion: Organizations should consider what evaluation objectives are planned and how they will be measured for a mock-up evaluation to determine if VR is appropriate.

## 1. Introduction

Designing or renovating a built environment for healthcare is a complex process that is riddled with challenges and opportunities. Patient and staff outcomes have been linked to healthcare environment design in more than 5000 citations [1].

The process used to design healthcare facilities is evolving to incorporate and produce learnings in evidence-based design. The Center for Health Design defined evidence-based design as “the process of basing decisions about the built environment on credible research to achieve the best possible outcomes.” [2]. Often the research comes from previous studies. In some cases, the data might not exist, or a design team might want to gather data which is specific to their planned design. Physical and VR mock-ups are increasingly being used as mechanisms to test or gather feedback and data on design concepts [3]. Conducting simulation-based mock-up evaluations by having planned users enact scenarios within a mock-up allows design teams to evaluate how effectively the built environment will support the planned processes [4]. Design decisions can then be made which are informed by evidence-based data gathered from the scenario enactments to enhance quality and patient safety.

Despite the rapidly growing trend to use VR mock-ups for healthcare facility design, little is known regarding the overall value and validity of using VR to conduct mock-up evaluations. There are numerous calls for research examining the use of VR mock-ups [5,6]. The Health Quality Council of Alberta recently developed guidelines comparing VR to physical mock-ups [7]. Those guidelines are intended to guide hospital design teams to select the most appropriate mock-up type. This manuscript is intended to elaborate specifically on the use of VR mock-ups, their associated strengths and weaknesses, and summarize technological advances. In addition, this manuscript contributes VR specific data for organizations considering the use of VR mock-ups.

### 1.1. Virtual Reality (VR)

VR mock-ups most commonly fall into three types which include passive, exploratory, and interactive [8,9]. In passive VR mock-ups, the participant remains stationary, and the VR world moves around them. In exploratory VR, the participant is able to move around within a stationary VR environment. Interactive VR mock-ups allow the participant to move around within the VR environment while also being able to manipulate and interact with the virtual world. These are also referred to as fully immersive virtual environments [10,11]. The latter is achieved through the use of a head-mounted display, where participants wear a visual display on their head and over their eyes [11,12]. A head-tracking device allows continuous updates to be provided on the visual display based on the location and orientation of the user’s head and hands as they interact with the virtual environment.

The use of VR technologies has been rapidly expanding over the past two decades and is expected to become an $80 billion market by 2025 [13]. To highlight only a couple healthcare examples, the use of VR has demonstrated effectiveness for use with stroke rehabilitation outcomes [14,15], as well as surgical training to perform a laparoscopy [16] and bronchoscopy [17]. VR is also more commonly being used as a tool in architectural design, initially being used in the design of courthouses [18,19], and later for healthcare environments such as hospital patient rooms [2,6,20,21,22], operating rooms [5,23], preoperative rooms [24], intensive care units [19], and hospital lobbies [18]. Many of these previous examples allowed users to perform walkthroughs of the space and provide feedback. Very few allowed interaction capabilities. Dunstan and colleagues used a virtual environment which allowed participants to make furniture and equipment reconfigurations, open doors to inspect clearance requirements, and adjust lighting levels to view the effects of both internal and external lighting sources and include audio recordings to enhance environmental realism [2,17].

Practitioners and researchers have cited numerous advantages of using VR mock-ups over physical mock-ups. These include enhanced ability to visualize design challenges and solutions, including the potential to simultaneously examine multiple design options. Consequently, design teams are more informed, enabling accelerated decision-making [5,18,25,26]. Some researchers have argued that using VR can decrease costs and time requirements in comparison to using physical mock-ups [7,21,27], however, others have noted that VR equipment is expensive [28], and the programming required to make the environment interactive is time-consuming and costly [2,10]. Design teams have been advised to measure the return on investment (ROI) before engaging in VR [18]. Perceptual challenges have been noted including the participants limited peripheral vision when viewing virtual environments (60 degrees of visibility) [7]. More recent technologies have added peripheral vision capabilities to address this limitation. Another perceptual challenge is the lack of haptic feedback (i.e., no physical cues if you hit a wall), and although technologies have solutions which allow haptic feedback, they are often not used in VR mock-ups [10]. The lack of perceived realism and immersion has been highlighted as an essential challenge when creating VR environments in a recent literature review [10]. The authors of the review put forward a suggested approach for conducting design research in VR environments, which includes designing task-based scenario and determining appropriate evaluation methods (qualitative and quantitative).

### 1.2. Scenario Enactment within VR Mock-Ups

The Health Quality Council of Alberta developed the Simulation-based Mock-up Evaluation Framework [3] which describes the process to conduct full scale mock-up evaluations using physical mock-ups. The Facilities Guidelines Institute has promoted the framework on their website [29]. The Canadian Standards Association has included it in the Canadian Health Care Facilities standard (CSA Z-8000) [30]. Organizations in both Canada [31] and the United States [32] have used the framework to evaluate operating rooms. The framework builds upon prior simulation-based mock-up evaluations used to evaluate hybrid operating theatres [33,34], ICU patient rooms [35], and assisted living resident suites [36]. Although the framework currently only describes the use of physical mock-ups, recent and anticipated advancements in VR capabilities make this technology a viable option to conduct a simulation-based mock-up evaluation.

Numerous successes have been demonstrated using physical mock-ups. For example, incorporating simulation-based mock-up evaluations during the design process resulted in a net savings estimated at $1.7 million [37]. Improvements in patient safety [27,29], staff efficiency [38], and room utilization [27] have also been realized. It is anticipated that VR mock-ups could provide the same successes. In addition, VR mock-ups have the potential to overcome some of the known limitations of physical mock-ups such as the costs and time to construct them, space requirements to house them, city permits to build them, and the difficulties adjusting them to reflect design changes made [7]. For these reasons, VR technologies are becoming more viable as an alternate or to complement the use of physical mock-ups.

Coupled with the potential benefits from using VR, there has been some recognition that scenario enactments and interaction capabilities requires specialized equipment [2]. Others have noted that simulations with multiple people are not possible at this point [7], yet it is a critical element to create the sense that multiple people are working together in a shared space, ultimately enhancing environmental realism and enabling collaborative work [39]. Furthermore, to fully assess how well the designed environment will support planned processes, as conducted in a simulation-based mock-up evaluation, it becomes critical to have multiple people simultaneously “working” in the VR environment. Software advances, such as Unity, now allow multiple participants to simultaneously interact within a virtual environment, making this technological advancement ripe for adoption by those interested to test drive a design prior to construction.

Given the benefits and limitations of VR, this study aimed to examine the overall value and validity of using VR mock-ups to conduct a simulation-based mock-up evaluation. ROI as well as stakeholder perceptions were included as measures of value. Validity included predictive validity as well as measured enhancements to quality and patient safety resulting from design modifications to the VR mock-up.

## 2. Materials and Methods

A post-occupancy evaluation (POE) is a structured approach to evaluate the performance of a new or existing facility when it is fully operational [30]. An existing medication room was examined through a POE to identify opportunities to enhance quality and patient safety. A VR mock-up was then developed replicating the design of the existing medication room. Data from the POE was used as a baseline to see if conducting a simulation-based mock-up evaluation within a VR mock-up could identify the same opportunities. More specifically, the intent was to assess the accuracy of data gathered from a simulation-based mock-up evaluation within a VR mock-up. The POE involved conducting 11 interviews with current users of the medication room (eight nurses and three pharmacy technicians) as well as video analysis of observational data collected over 12 h during peak medication room usage times over two days. Video recordings were captured from four camera angles. All participants gave written consent for the video and audio recordings. Video analysis involved conducting link analyses to assess workflow and coding instances of bumps, impediments, interruptions, task completion times, searching behaviours and selection errors.

A medication room was selected as the subject of this evaluation because of the safety implications that surround medication preparation and because medications are prepared and administered to almost every patient across nearly all healthcare settings. Medication errors are the most common type of medical error [40]. Most medication errors (58%) occur during administration (providing patients with a prescribed medication) [41]. Although nearly half of medication prescribing errors are detected and intercepted before reaching the patient, only two percent of errors occurring at the administration stage are intercepted [42]. In Alberta, Canada, where this study was conducted, an external review of an adverse event involving a medication substitution error resulting in two patient deaths recommended that the “adequacy of areas for medication preparation in patient care areas be assessed and renovations undertaken where necessary” (p. 81, [43]).

**Figure 1:**
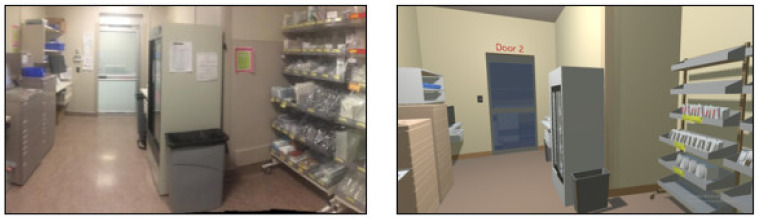
Existing medication room (**left**) and VR medication room mock-up (**right**).

VR mock-ups (fully interactive virtual environments) were developed by a VR software company (SimInsights) to replicate the design of the existing medication room evaluated through the POE (Figure 1). The VR mock-up was developed using an AutoCAD 2D layout of the room. Photos of the room, equipment, and objects were also used to enhance details of the virtual environment. The VR mock-up was programmed to be fully interactive, allowing up to four individuals to be simultaneously immersed and interact using four HTC Vive headsets and controllers. The VR mock-up included an interactive automated medication dispensing cabinet, Wi-Med carts and a pharmacy cart which were maneuverable and operable, interactive electronic medication administration records, a fridge stocked with IV bags, and supply cabinets. The head-mounted display worn by participants included speakers and a microphone enabling participants to communicate with each other. All of these features enhanced realism intending to allow participants to feel fully immersed and present in the virtual environment.

A simulation-based mock-up evaluation was conducted within the VR mock-up. This involved having end-users’ participants enact realistic scenarios within the VR mock-up while behavioural data was collected. Four different scenarios were enacted within the VR mock-up.

The first scenario involved four participants independently working one at a time in the medication room. Nurse 1 and Nurse 2 prepared multiple medications for a morning medication pass consisting of four patients. Nurse 3 prepared an urgently needed (STAT) medication for a single patient. Pharmacy Technician 1 stocked multiple medications and supplies into the medication room. The second scenario involved two individuals simultaneously working together in the medication room (Nurse 2 and Pharmacy Technician 1). The third scenario involved three individuals simultaneously working together (Nurse 1, Nurse 3 and Pharmacy Technician 1). The fourth scenario involved the enactment of all four roles simultaneously (Nurses 1–3 and Pharmacy Technician 1). The full scenarios can be found in the Supplementary Materials. The same four scenarios were also enacted by the same participants in a revised layout of the medication room (proposed layout) which incorporated design modification to address the opportunities to enhance quality and patient safety identified through the POE.

The entire process was repeated over three days (four scenarios × two room layouts × three days) with different participants each day. Over the three days, 15 participants (12 nurses and three pharmacy technicians) enacted various roles in the scenario enactments. All participants gave written consent prior to participating.

In addition to developing the VR environment, SimInsights was also contracted to program software (HyperMock) which allowed for automated data collection and analysis of behavioral data. The measures selected for analysis were based on the Simulation-based Mock-up Evaluation Framework [4] as well as measure used in prior mock-up evaluations of healthcare facilities [31,33,34,35,36,37,38] and were adapted for applicability to a medication room. A manual data collection and analysis process is typically used with simulation-based mock-up evaluations, particularly when physical mock-ups are used. Manual data collection and analysis of video footage also occurred for the POE. Automated collection of scenario enactment data included the following measures and associated data definitions:

- Workflow (link analysis)—movement paths for each individual involved in the scenario enactments. These were transcribed onto a room layout diagram. Layering multiple link analyses allowed for visualization of room utilization, movement patterns, and the identification of high-traffic areas within the room;
- Bumps—instances of physical contact between two objects (people and/or equipment) that were not intended to make contact. The total number of bumps were examined as an indicator of congestion;
- Impediments—instances where an individual experienced an object or person that obstructed their path. More specifically, an impediment was defined as an instance where a path travelled between two objects was more than 20 per cent, and at least one meter, longer because that individual needed to go around a person or moveable object. Both the total number of impediments and those experienced while performing specific subtasks (i.e., accessing the sharps container to dispose of a used needle) were examined as indicators of congestion;
- Interruptions—instances where an individual’s attention, while performing a task, was diverted away from the task at hand by another person. The total number of interruptions were examined;
- Task completion times—the total time from when an individual entered the medication room mock-up until when they exited.

Survey data from scenario enactment participants, hospital design stakeholders, and recipients of POE recommendations captured subjective feedback regarding the POE and VR mock-up. A survey of scenario enactment participants was completed after every scenario enactment by each individual participating in the scenario enactment. A total of 13 roles were enacted across the four scenarios: four roles in scenario one, two roles in scenario two, three roles in scenario three, and four roles in scenario four. These scenarios were re-enacted within a second medication room layout (proposed design) which is described later. The post-scenario enactment surveys were completed after these scenario enactments as well. As such, 26 roles were enacted, and 26 surveys were completed each day. The entire process was repeated over three days. The post-scenario enactment survey inquired about the realism of the VR mock-up and scenarios enacted as well as feedback on various room design features. Forty-three hospital design stakeholders attended a VR presentation and demonstration to learn more about the project and VR capabilities. The stakeholders included members of the capital planning team (project manager and leadership), clinical leadership, architects, project managers of hospital construction companies, human factors specialists, process improvement consultants, and VR experts. Surveys were administered to the stakeholders after the presentation and demonstration inquiring about the perceived utility of VR mock-ups to inform decisions regarding room design. POE recommendation recipients consisted of the members of the Medication Management Committee at the acute care hospital where the POE occurred. This committee consisted of managers and directors from various hospital units, Pharmacy Services, and Patient Safety. The survey asked the recommendation recipients whether they thought (a) the recommendations would address the issues identified through the POE, (b) the recommendations were beneficial, and (c) if they intended to implement the POE recommendations.

Return on investment (ROI) was monetized using the Phillips ROI methodology and examined the monetary benefits versus costs [44]. The costs included creating the VR mock-ups as well as the automated data collection software, honorariums paid to participants, travel expenses, and lunch catering. An undeveloped section of the hospital was provided at no cost to conduct the evaluation. Monetary benefits were calculated as the cost avoidance of future renovations.

The POE and VR mock-up evaluations occurred after successfully completing the Alberta Research Ethics Community Consensus Initiative (ARECCI) screening tool [45].

## 3. Results

### 3.1. Survey Results

A post-scenario enactment survey was completed by every participant after every role they enacted. Each day, 26 roles were enacted, and the process was repeated over three days. In total, 78 surveys were completed (100 per cent response rate). Responses indicated that, on average, there was agreement or strong agreement from participants that the medication room and scenarios were realistic, the scenarios enacted represented realistic workflow, and participants believed they could effectively evaluate the room design and identify opportunities for improvement (Table 1). These findings are important because a realistic mock-up and scenario enactments are needed to properly test the planned environment with accurate workflows. In addition, participants felt they were able to contribute and improve the planned design.

Another survey was administered to both scenario enactment participants as well as the hospital design stakeholders. The survey was completed by the 15 scenario enactment participants (100 per cent response rate) and was completed at the end of the day capturing perceptions across all scenarios enacted. The stakeholder survey was completed by 20 of the 43 individuals who attended a VR presentation and demonstration (47 per cent response rate). Stakeholders asked if they could take the survey home to complete and email it back to the researcher. Many did not return their survey resulting in the lower response rate. Results indicated that, on average, there was agreement or strong agreement from participants and stakeholders that enacting scenarios within the VR mock-up allowed for accurate feedback regarding 13 of the 15 evaluation objectives listed, though they differed on which two objectives were rated lower (less than 4.00; Table 2). This finding highlights how both participants and stakeholders believe that VR mock-up evaluations provide accurate feedback across a diverse set of possible evaluation objectives.

The hospital design stakeholders were also asked to rate on a scale from 1 (strongly disagree) to 5 (strongly agree) the realism of the mock-up and the utility of the evaluation process for future projects. On average, there was agreement or strong agreement from stakeholders that the VR mock-up environment was realistic (average of 4.41) and that the information gathered from this evaluation method will be useful for future projects (average of 4.53).

### 3.2. Predictive Validity

Comparisons of post-occupancy evaluation (POE) data collected from the existing medication room to mock-up evaluation data collected from the VR mock-up included workflow (link analyses) and task completion times.

#### 3.2.1. Workflow

**Figure 2:**
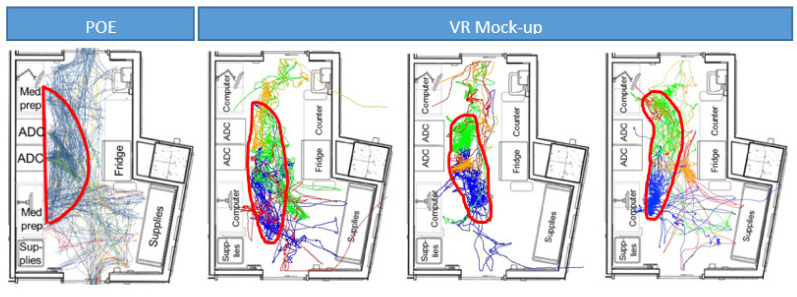
High traffic areas were observed in front the of the automated medication dispensing cabinet (ADC) in the real workflow captured from the POE (**left**) as well as through scenario enactment workflow captured from the VR mock-up evaluation (**right most 3**).

A link analysis was used to identify high-traffic areas based on observed workflows in the medication room and the VR mock-up (Figure 2). Although link analyses were created for all scenarios, only the three scenarios where four people worked simultaneously within the VR mock-up are displayed alongside the link analysis from the POE. The red circle highlights the area of the room with the highest volume of traffic. As noted, workflow patterns are fairly consistent between the POE and scenario enactments within the VR mock-up. Specifically, in all cases, the highest traffic area was located in front of the automated medication dispensing cabinet and the computer workstations used for medication preparation. This supports the notion that workflow data collection can be automated within a VR mock-up and is an accurate predictor of actual medication room workflow.

#### 3.2.2. Task Completion Times

Average task completion times for various equivalent tasks were calculated and comparisons were made between data gathered from the POE and the VR mock-up evaluation (Table 3). Results suggested that the differences ranged from 12–36 per cent, but on average the difference between actual versus simulated task completion times was 1% (SD = 32.5). This supports the notion that time-based measurement can be automated within a VR mock-up and provides a reasonable estimate of real task completion times.

### 3.3. Enhancing Quality and Patient Safety

Opportunities to enhance the existing medication room design were identified through the POE. These learning opportunities were used to assess whether incorporating the design modifications would enhance quality and patient safety as measured through behaviors and perceptions.

#### 3.3.1. Post-Occupancy Evaluation Learnings

Qualitative and video analysis data from the POE uncovered specific issues that medication room users experienced when working in the medication room. The first issue pertained to the location of the medication dispensing cabinet and how end-users working at the cabinet hindered workflow for other medication room users throughout the rest of the medication room, and specifically between the medication preparation areas and the medication supplies. The second issue was that the wireless medication cart often blocked access to the only sharps container when preparing medications. The third issue was that patient specific bins were stored in two locations. Retrieving bins from both locations resulted in congestion, which was exacerbated by the medication cabinet location. Three design-based recommendations were developed to address the identified issues and were intended to enhance specific anticipated outcomes related to quality and patient safety. The recommendations and anticipated outcomes are listed in Table 4.

#### 3.3.2. Testing Enhancements through Behavioural Data

**Figure 3:**
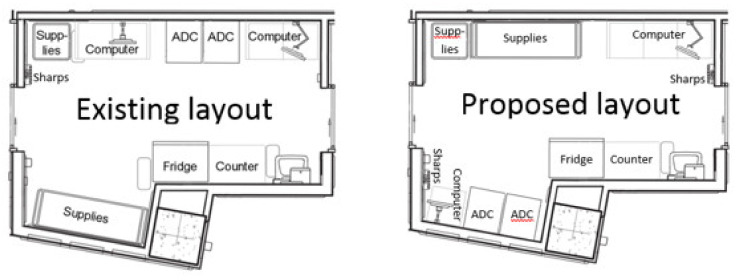
Two medication room layouts were evaluated: the existing (**left**) and the proposed (**right**).

Two different medication room layouts were created as VR mock-ups. The first layout was an exact replica of the existing medication room design (existing layout); the second layout incorporated design modification as per the POE recommendations (proposed layout). Both layouts are shown in Figure 3. Enhancements to quality and patient safety were evaluated by comparing data specific to the anticipated outcomes in both layouts.

Where measurable, the results supported that the proposed design changes would results in measurable differences in quality and patient safety between the existing and proposed layouts (Table 5). Specifically, the data supported that the design changes resulted in the following anticipated outcomes:

- Fewer interruptions;
- Less congestion (as measured by both bumps and impediments);
- Better access to the fridge (when measured by impediments);
- Less medication preparation time;
- Less congestion when accessing the sharps container, and
- Less congestion when accessing patient bins

Some of the anticipated outcomes and units of measurement were identified after the scenarios were enacted (post-hoc) through the scenario enactment debriefing session with scenario participants. Consequently, the procurement process for the VR software did not specify that the VR vendor should program the software for automated data collection of these measures. As a result, the accuracy of these measures was not assessed. For example, comparing where Wi-Med and pharmacy carts were stored within the room during scenario enactments, and how this differed between the two layouts was identified as being important during the debriefing sessions. Identifying this possible measure occurred after procuring the VR software, and therefore data regarding cart placement in the VR mock-up could not be collected. In contrast, these types of data collection changes can be incorporated when manually coding video data. It is fairly common to change or revise planned measures based on debriefing feedback. The same was true for measuring bumps that occurred while specifically accessing the fridge. Although the VR software was programmed to capture bump data, the data could not be filtered to only include bumps involving the fridge. Additionally, patient specific bins were used to store medications specific to a particular patient after retrieving these medications from the automated medication dispensing cabinet. These bins were labelled with a patient identifier. During the debriefing session, one nurse noted that they accidentally selected a bin for the wrong patient. If left unnoticed, there is an increased likelihood that medications placed in that bin could be administered to the wrong patient. Collecting data pertaining to bin selection errors was not programmed for automated data collection and therefore the accuracy of this measure was not assessed. These few examples highlight the importance of having a clear understanding of what measures will be used before procuring or programming the VR software or including a plan for iterative programming based on debriefing feedback.

Despite being included in the procurement specifications, the VR vendor indicated it was not possible to program software which would identify searching behaviours. Storing all patient bins together was intended to make it easier to find patient bins as measured through searching behaviours. This included both the number of instances and the total time spent looking in the wrong locations. When manually coding these behaviours, it is possible to assess these, however, neither was possible to automate with the VR software. Therefore, it was not possible to assess these as part of the VR mock-up evaluation.

#### 3.3.3. Testing Enhancements through Survey Data

Post-scenario enactment survey data supported the behavioural data. Scenario enactment participants were asked to rate their level of agreement with various design outcomes related to quality and patient safety on a scale from 1 (strongly disagree) to 5 (strongly agree). Results suggested that participants liked the proposed layout more than the existing layout (Table 6). Additionally, they perceived the proposed layout to be less congested and provided better access to the electronic medication administration record. The difference in their perceived access to medications and supplies was not significant.

### 3.4. Return on Investment (ROI)

ROI was calculated by determining the monetary benefits versus the monetary cost of conducting a simulation-based mock-up evaluation within a VR mock-up. Monetary costs included creating the VR mock-ups as well as the automated data collection software, travel expenses, honorariums paid to scenario enactment participants, and lunch catering. In total, the hard costs were CAD 84,838.

In addition, two human factors specialists spent 152 h conducting the VR mock-up evaluations. To monetize this amount, an average hourly rate for a human factors specialist, including benefits (CAD 72.87 per hour), plus general and administrative expenses for the organizations (CAD 20 per hour) was used to calculate soft costs. Combining hard and soft costs, the VR mock-up evaluation cost CAD 99,016.

Monetary benefits were calculated as the cost avoidance of future renovations. The average cost to renovate a medication room, as per the capital planning department where this evaluation occurred, ranges from CAD 75,000 to CAD 100,000 for a minor renovation, and ranges from CAD 250,000 to $300,000 for a typical medication room renovation. In accordance with ROI principles, the most conservative renovation cost was used to calculate ROI to produce the most conservative estimate. Given there were eight medication rooms with the same design at the facility where the POE occurred, the cost to renovate all medications was estimated at CAD 600,000 (eight rooms × CAD 75,000).

ROI is a financial metric which is calculated using the project benefits and costs. When presented as a per cent ROI, it is calculated using the following formula:

(1) $$\mathrm{ROI}\;(\%)=\frac{\mathrm{Net}\;\mathrm{Project}\;\mathrm{Benefits}\;(\mathrm{project}\;\mathrm{benefits}-\mathrm{project}\;\mathrm{costs})}{\mathrm{Project}\;\mathrm{Costs}}\times 100=\frac{\$600,000-\$99,016}{\$99,016}\times 100=506\%$$

This suggests that a VR mock-up can produce a 506% ROI. Stated another way, CAD 5.06 can be saved for every dollar invested in a VR mock-up, beyond the initial investment. The Phillips ROI methodology used for this calculation produces the most conservative ROI estimate. The potential savings are likely greater, especially in cases involving larger scope renovations, use of advanced VR technologies, or when evaluating room templates such as patient care rooms.

## 4. Discussion

Using VR to conduct a simulation-based mock-up evaluation of healthcare facility designs can produce a positive and substantial return on investment (ROI). A variety of measures can be accurately evaluated and are predictive of actual workflows and behaviours that will eventually occur once constructed, occupied, and used for healthcare processes. For instance, VR is capable of accurately capturing data to assess how design changes will affect:

- High-traffic areas within the room;
- Task completion times;
- The frequency of staff interruptions during critical tasks;
- Access to frequently or urgently needed supplies or devices, and
- Room congestion.

Although most measures assessed were found to be accurate predictors, identifying and qualifying searching behaviors was not. As such, organizations should consider what evaluation objectives are planned and how they will be measured for a mock-up evaluation to determine if VR is appropriate.

The ability to automate the data collection process is one of the distinct advantages of using a VR mock-up. The VR mock-ups used in this evaluation required pre-programming of each planned measure. Exploratory or post-hoc measures identified after the scenario enactments would have required additional software programming funds as well as time, and therefore were not assessed in this study. Examples of post-hoc measures from this evaluation that were not assessed included plotting cart storage locations, identifying bumps associated with specific items in the room, and selection errors. This highlights another reason why it is important to have a clear understanding of the evaluation objectives before initiating a VR mock-up evaluation.

Expediently delivering results following scenario enactments within the mock-up is often an important request from healthcare design teams. Automated data collection and analysis produces much faster turnaround times compared to manual data collection and analysis which is common with simulation-based mock-up evaluations in physical mock-ups. Being aware of these opportunities and limitations when using VR mock-ups will help organizations consider the most cost-effective mock-up type that (1) provides an appropriate level of fidelity to assess the design considerations being tested and (2) allows collection and analysis of data measures that accurately assesses the evaluation objectives of interest to the design team.

Although participants perceived the scenarios enacted as being realistic, technological limitations may have limited realism. For example, haptic feedback was not provided to participants. In other words, when a participant bumped into something, such as a fridge, they did not feel the bump. Moreover, this VR evaluation required a tethered connection from the participant’s head-mounted display (HMD) to the computer, which somewhat hindered participant’s ability to move freely during scenario enactments. Despite these technological limitations, the evaluation was still capable of providing accurate and valid data to assess measures related to quality and patient safety.

Technological advances over the last few years offer even greater value for practitioners in terms of expanded range of studies, reduced cost, increased performance and fidelity, richer data collection, and faster analytics. Greater ROI’s, decreased time requirements, and enhanced validity is expected. The next sections describe some of the technological advances which have occurred since this evaluation was conducted. These advancements have created new opportunities for organizations wanting to integrate simulation-based mock-up evaluations using VR technology into their healthcare facility design process.

### 4.1. VR Head Mounted Displays (HMDs) and Accessories

Prices of VR HMDs have fallen substantially with the launch of Oculus Quest 2 (299 USD) in 2021. Quest 2 also greatly simplified the set-up as it does not require external trackers to be installed, and there is no wired connection to the PC or Mac necessary. Similar low-cost untethered HMDs are available from other manufacturers as well (e.g., Pico). Simplicity and low price mean that many more participants can be included in studies. For example, a small box containing the headset can be shipped to participants via mail.

Other vendors have made available expensive VR headsets that push the limits of performance. Varjo VR HMD provides eye-resolution experience, practically indistinguishable from reality. For some tasks that require very high resolution (e.g., threading a needle), the low resolution of low-cost VR headsets prove to be insufficient.

The visual modality of VR HMDs is supplemented by an increasing number of accessories. For tactile feedback, glove accessories available from Haptx, VRGluv and other vendors, bring the sense of touch to VR. Omnidirectional treadmills are available to simulate walking around, thus enabling studies to be conducted in settings where physical space is limited.

### 4.2. Authoring Software

Creators of VR simulations can choose from game engines such as Unity and Unreal Engine or use authoring tools such as HyperMock. While the former provides vast flexibility in defining the nuances of the simulations using powerful programming languages (C# and C++, respectively), they also require multidisciplinary teams consisting of skilled software developers and 3D artists. In contrast, authoring tools provide a low cost and efficient alternative enabling subject matter experts to quickly provide fully functional, high fidelity 3D simulations that, in many cases, sufficiently capture the task-characteristics necessary for generating valid and reliable data. When an authoring tool suffices, the time and cost of VR simulation, data collection and analytics can be reduced substantially, often by orders of magnitude.

### 4.3. Data Collection, Machine Learning and Analytics

Many VR HMDs are available with built-in or add-on eye tracking capability, which provides gaze and pupillometry data. From this dataset, attention and cognitive load information can be extracted if required. Machine learning techniques have developed rapidly in recent years, providing powerful methods for understanding the behaviours of human participants. For example, automated speech recognition enables capture of dialogue between human subjects during the VR experience. Deep learning techniques make it possible to understand the intent behind the spoken utterances of the users. Together these two capabilities can be used to semantically understand the participants’ dialogue during scenario enactment. This can be useful for coding certain behaviours such as access issues and interruptions. When human factors studies are conducted with physical mock-ups and computer vision techniques can be applied to automate the behaviour coding of video capture data.

## 5. Conclusions

The findings suggest that the use of VR mock-ups can produce a 506% return on investment (ROI). Stated another way, CAD 5.06 can be saved for every dollar invested in a VR mock-up, beyond the initial investment. As this is based on one case study, future uses of VR to conduct a simulation-based mock-up evaluation can anticipate a different ROI depending on the room(s) evaluated, technology used, and other factors. Furthermore, evidence-based data collected through the VR mock-up accurately assessed several measures: workflow (link analysis), bumps, impediments, interruptions, and task completion times. Searching behaviours were not possible to capture using the VR software. Collecting data pertaining to selection errors and equipment placement were identified after procuring the VR software and therefore the accuracy of these measures was not assessed. Scenario enactment participants rated the VR mock-up and the scenarios enacted to be very realistic. Current and anticipated technological advances offer even greater value for practitioners interested to optimize quality and safety as part of the healthcare facility design process.

## Figures and Tables

**Table 1:** Post-scenario enactment survey responses. On a scale of 1 (strongly disagree) to 5 (strongly agree), participants were asked: “Based on this scenario enactment, please rate your level of agreement with the following statements”.

Survey Question	Average Participant Response
The medication room mock-up was realistic.	4.61
This scenario was realistic.	4.61
The enactment of this scenario represented realistic workflow.	4.60
I was able to effectively evaluate the design of this room.	4.52
I am able to identify opportunities to improve the design.	4.47

**Table 2:** Survey responses from scenario enactment participants and hospital design stakeholders. On a scale of 1 (strongly disagree) to 5 (strongly agree), respondents were asked: “This evaluation method would allow me to provide accurate feedback regarding”.

Evaluation Objectives	Average Participant Response	Average Stakeholder Response
Unit configuration	4.77	4.40
Room size	4.14	3.88
Design or design feature comparisons (e.g., compare room layouts)	4.64	4.56
Space requirements for equipment or processes	3.93	4.38
Access to the patient and/or equipment	4.29	4.25
Patient/family spaces and experiences	4.00	4.07
Patient transport routes to and from the room	3.50	4.25
Room configuration	4.64	4.56
Furniture, fixtures, and equipment placement	4.43	4.63
Furniture, fixtures, and equipment usability	4.36	4.25
Visibility of patient, monitors, supplies, and/or equipment	4.07	4.67
Supply placement	4.50	4.60
Adverse events	4.08	4.14
Work flows and processes	4.64	4.20
Team functioning/performance	4.36	3.93

**Table 3:** Task completion times (averaged) for various tasks as measured during the POE and in the VR mock-up evaluation.

Task	Average Task Completion Time (Min:Sec)		Per Cent Difference
	POE	VR	
Medication stocking	5:39	6:19	12%
Medication preparation (single STAT medication)	1:28	1:51	26%
Medication preparation (morning medication pass)	7:50	5:03	−36%
Average			1%

**Table 4:** Recommended design modifications and associated anticipated outcomes identified through the POE.

Recommendations	Anticipated Outcomes
Switch the location of the medication supplies with the automated medication dispensing cabinet and medication preparation area.	○ reduce the number of interruptions ○ more effective cart storage ○ reduce congestion ○ better access to the fridge ○ reduce time for medication preparation
2. Include a sharps container within arm’s reach of medication preparation areas.	○ reduce congestion when accessing the sharps container
3. Store all patient bins together.	○ make it easier to find patient bins ○ reduce congestion when accessing patient bins ○ reduce likelihood of selecting the wrong patient bin

**Table 5:** Comparison of anticipated outcomes between the existing and proposed layout based on data collected through the VR mock-up evaluation.

Anticipated Outcomes	Unit of Measurement	Existing Layout	Proposed Layout
Reduce the number of interruptions	Occurrences of interruptions	50	6
More effective cart storage	Cart placement (Wi-Med and pharmacy)	VR not programmed for measurement	
Reduce congestion	Occurrences of bumps	136	103
	Occurrences of impediments	54	49
Better access to the fridge	Occurrences of bumps while accessing the fridge	VR not programmed for measurement	
	Occurrences of impediments while accessing the fridge	2	1
Reduced time for medication preparation	Average time to prepare a single/stat medication (mm:ss)	1:51	1:09
Reduce congestion when accessing the sharps container	Occurrences of impediments while accessing the sharps container	5	1
Make it easier to find patient bins	Occurrences of searching for patient bins	Not possible to program VR for measurement	
	Time searching for patient bins	Not possible to program VR for measurement	
Reduce congestion when accessing patient bins	Occurrences of impediments while accessing patient bins	15	9
Reduce likelihood of selecting the wrong patient bin	Occurrences of selecting the wrong patient bin	VR not programmed for measurement	

**Table 6:** Comparison of survey responses from scenario enactment participants between the existing and proposed layout.

Anticipated Outcomes	Mean (SD)		T (df)	*p*-Value
	Existing Layout	Proposed Layout		
I liked the design of this room	3.49 (1.26)	3.89 (0.91)	−1.70 (73)	0.047 *
The room felt congested	3.83 (2.21)	3.14 (2.34)	1.95 (70)	0.03 *
I could easily access medications and supplies	3.58 (1.56)	3.95 (1.24)	−1.32 (72)	0.09
I could easily access the electronic medication administration record	4.17 (1.25)	4.64 (0.53)	−1.91 (56)	0.03 *

* Indicates statistically significant difference.

## Data Availability

Data can be found by contacting the corresponding author of this study.

## Supplementary Materials

The following are available online at https://www.mdpi.com/article/10.3390/ijerph182111250/s1, Simulation Scenario Roles.
