# Supplementary material for: Using Virtual Reality (VR) Mock-Ups for Evidence-Based Healthcare Facility Design Decisions
Source: Int J Environ Res Public Health. 2021 Oct 26;18(21):11250. doi: 10.3390/ijerph182111250 (PMC8583497; doi:10.3390/ijerph182111250)
Supplement: Supplementary file 1 [file ijerph-18-11250-s001.zip › ijerph-1413444-supplementary.pdf]

Supplementary Material: Simulation Scenario Roles

Scenario: Nurse 1

You have just come on shift and are preparing to administer your morning medication pass. You have four patients assigned to you (Patient A, Patient B, Patient C and Patient D). To prepare for your medication pass, you will be taking your Wi-Med cart into the medication room, gathering the blue patient specific bins for your patients, filling the patient specific bins with needed medications and supplies, and then placing the patient specific bins into your Wi-Med cart. Once you have gathered meds and supplies for all of your patients, you exit the medication room through the same doors you entered (door 1).

To open the med room door, touch the card reader (black box beside the doors). Place your hands under the faucet, soap, or hand sanitizer to indicate usage. To view the electronic medication administration record (eMAR) touch any computer screen, except for the screen on the Automated Dispensing Cabinet (ADC). Touch it again to view the next medication ordered. The eMAR will also indicate where medications are stored. To retrieve medications, touch the ADC computer screen which will direct you to an ADC drawer or the fridge. Injectable medications can be prepared by gathering the medications and supplies needed, placing them onto a work surface and holding the progress ball (symbolizes time typically used preparing the IV bag). Supplies are stored on the supply shelf and in the drawer below the med prep areas with the computers. When preparing pantoprazole, assume the vial is premixed and the patient already has a primary line running in their room so you only need the secondary line. You don't need to get the AeroChamber for the inhaler because it is stored in the supply room.

Nurse 1 Tasks

- enter medication room from door 1 with a Wi-Med cart
- use the hand sanitizer
- get patient specific bins for Patients A,B,C,D and place bins onto any work surface
  - NOTE: Blue bins for patient are above both med prep areas. They are labelled "Patient A", etc.

Prepare Medications for Patient A

- use any computer to identify which medications are needed for Patient A
- gather medications for Patient A and put them into the bin for Patient A
  - dalteparin inj 5,000 unit(s) SUBCUTANEOUSLY q24h, start at 08:00 (from ADC)
  - KCl 40 mmol in 0.9% NaCl infusion (Known as: potassium chloride 40 mmol in 0.9% NaCl infusion) 1,000 mL IV <Continuous>, start at 08:00 at 100 mL/hour (from large supply shelves)
  - metoPROLOL tab 50 mg PO bid, start at 08:00 (from ADC)
  - ibuprofen tab 800 mg PO daily, start at 08:00 (from ADC)
- put Patient A's bin into Wi-Med cart

Prepare Medications for Patient B

- use any computer to identify which medications are needed for Patient B
- gather medications for Patient B and put them into the bin for Patient B
  - acetaminophen tab 650 mg PO daily, start at 08:00 (from ADC)
  - thiamine tab 100 mg PO daily, start at 08:00 (from ADC)
  - metoPROLOL tab 50 mg PO bid, start at 08:00 (from ADC)
  - amoxicillin / clavulante tab 875 mg / 125mg PO bid, start at 08:00 (from ADC)
  - pantoprazole inj 40 mg IVPB daily, start at 08:00, requires secondary line only (from ADC, in 100ML 0.9% NaCl)
- gather the following for preparation of Pantoprazole
  - medication added label, orange (from drawer below med prep area)
  - IV line label, white (from drawer below med prep area)
  - alcohol swabs (from drawer below med prep area)
  - blunt fill needle (from large supply shelves OR drawer below med prep area)
  - 20 mL syringe (from large supply shelves)
  - 100 mL 0.9% Sodium Chloride injection USP (from large supply shelves)
  - secondary medication set (from small supply shelves)

- prepare Pantoprazole IV bag
  - hold progress ball at work surface until task is completed
  - throw out garbage
  - put syringe and needle into sharps bin
  - put IV bag and line label into patient bin
  - put secondary medication set onto Wi-Med cart
- put Patient B bin into Wi-Med cart

Prepare Medications for Patient C

- use any computer to identify which medications are needed for Patient C
- gather medications for Patient C on put them into the bin for Patient C
  - diclofenac 2.32% gel Apply TOPICALLY for back pain bid, start at 08:00 (from ADC)
  - salbutamol inhaler 1 puff(s) INHALED daily, start at 08:00 (from ADC)
  - amoxicillin / clavulante tab 875 mg / 125mg PO bid, start at 08:00 (from ADC)
  - thiamine tab 100 mg PO daily, start at 08:00 (from ADC)
- put Patient C's bin into Wi-Med cart

Prepare Medications for Patient D

- use any computer to identify which medications are needed for Patient D
- gather medications for Patient D on put them into the bin for Patient D
  - ampicillin inj 1 g IVPB once, start at 08:00, in 100 mL mini-bag plus (from fridge [entered into ADC first, ADC direct to bin in fridge])
  - salbutamol inhaler 1 puff(s) INHALED daily, start at 08:00, via aerochamber, (from ADC)
  - acetaminophen tab 650 mg PO daily, start at 08:00 (from ADC)
  - piperacillin / tazobactam inj (known as TAZOCIN inj) 4.5 g IVPB once, start at 08:00, in 100ML D5W, (from fridge [entered into ADC first, ADC direct to bin 6 in fridge])
- put Patient D's bin into Wi-Med cart
- use the hand sanitizer
- exit with Wi-Med through door 1

Scenario: Nurse 2

You have just come on shift and are preparing to administer your morning medication pass. You have four patients assigned to you (Patient E, Patient F, Patient G and Patient H). To prepare for your medication pass, you will be taking your Wi-Med cart into the medication room, gathering the blue patient specific bins for your patients, filling the patient specific bins with needed medications and supplies, and then placing the patient specific bins into your Wi-Med cart. Once you have gathered meds and supplies for all of your patients, you exit the medication room through the same doors you entered (door 2).

To open the med room door, touch the card reader (black box beside the doors). Place your hands under the faucet, soap, or hand sanitizer to indicate usage. To view the electronic medication administration record (eMAR) touch any computer screen, except for the screen on the Automated Dispensing Cabinet (ADC). Touch it again to view the next medication ordered. The eMAR will also indicate where medications are stored. To retrieve medications, touch the ADC computer screen which will direct you to an ADC drawer or the fridge. Injectable medications can be prepared by gathering the medications and supplies needed, placing them onto a work surface and holding the progress ball (symbolizes time typically used preparing the IV bag). Supplies are stored on the supply shelf and in the drawer below the med prep areas with the computers. When preparing pantoprazole, assume the vial is premixed and the patient already has a primary line running in their room so you only need the secondary line. You don't need to get the AeroChamber for the inhaler because it is stored in the supply room.

Nurse 2 Tasks

Enter medication room (1 minute after Nurse 1 if applicable) from door 2 with a Wi-Med cart

- NOTE: Nurse 1 will still be preparing medications

Wash hands at the sink

Get patient specific bins for Patients E,F,G,H and place bins onto any work surface

- NOTE: Blue bins for patient are above both med prep areas. They are labelled "Patient E", etc.

Prepare Medications for Patient E

- use any computer to identify which medications are needed for Patient E
- gather medications for Patient E and put them into the bin for Patient E
  - dalteparin inj 5,000 unit(s) SUBCUTANEOUSLY q24h, start at 08:00 (from ADC)
  - KCl 40 mmol in 0.9% NaCl infusion (known as: potassium chloride 40 mmol in 0.9% NaCl infusion) 1,000 mL IV <Continuous>, start at 08:00 at 100 mL/hour, (from large supply shelves)
  - metoPROLOL tab 50 mg PO bid, start at 08:00 (from ADC)
  - ibuprofen tab 800 mg PO daily, start at 08:00 (from ADC)
- put Patient E's bin into Wi-Med cart

Prepare Medications for Patient F

- use any computer to identify which medications are needed for Patient F
- gather medications for Patient F and put them into the bin for Patient F
  - acetaminophen tab 650 mg PO daily, start at 08:00 (from ADC)
  - thiamine tab 100 mg PO daily, start at 08:00 (from ADC)
  - metoPROLOL tab 50 mg PO bid, start at 08:00 (from ADC)
  - amoxicillin / clavulante tab 875 mg / 125mg PO bid, start at 08:00 (from ADC)
  - pantoprazole inj 40 mg IVPB daily, start at 08:00, requires secondary line only, (from ADC, in 100ML 0.9% NaCl)
- gather the following for preparation of Pantoprazole
  - medication added label, orange (from drawer below med prep area)
  - IV line label, white (from drawer below med prep area)
  - alcohol swabs (from drawer below med prep area)
  - blunt fill needle (from large supply shelves OR drawer below med prep area)
  - 20 mL syringe (from large supply shelves)
  - 100 mL 0.9% Sodium Chloride injection USP (from large supply shelves)
  - secondary medication set (from small supply shelves)
- prepare Pantoprazole IV bag
  - hold progress ball at work surface until task is completed
  - throw out garbage
  - put syringe and needle into sharps bin
  - put IV bag and line label into patient bin
  - put secondary medication set onto Wi-Med cart
- put Patient F bin into Wi-Med cart

Prepare Medications for Patient G

- use any computer to identify which medications are needed for Patient G
- gather medications for Patient G on put them into the bin for Patient G
  - diclofenac 2.32% gel Apply TOPICALLY for back pain bid, start at 08:00 (from ADC)
  - salbutamol inhaler 1 puff(s) INHALED daily, start at 08:00 (from ADC)
  - amoxicillin / clavulante tab 875 mg / 125mg PO bid, start at 08:00 (from ADC)
  - thiamine tab 100 mg PO daily, start at 08:00 (from ADC)
- put Patient G's bin into Wi-Med cart

Prepare Medications for Patient H

- use any computer to identify which medications are needed for Patient H
- gather medications for Patient H on put them into the bin for Patient H
  - ampicillin inj 1 g IVPB once, start at 08:00, in 100 mL mini-bag plus (from fridge [entered into ADC first, ADC direct to bin in fridge])
  - salbutamol inhaler 1 puff(s) INHALED daily, start at 08:00, via AeroChamber (from ADC)
  - acetaminophen tab 650 mg PO daily, start at 08:00 (from ADC)
  - piperacillin / tazobactam inj (known as TAZOCIN inj) 4.5 g IVPB once, start at 08:00, in 100ML D5W (from fridge [entered into ADC first, ADC direct to bin 6 in fridge])
- put Patient H's bin into Wi-Med cart

Wash hands at the sink

Exit with Wi-Med through door 2

Scenario: Nurse 3

You have a patient who has is vomiting and plan to administer Ondansetron quickly to avoid potential complications with a fresh post-operative abdominal incision. You enter the medication room, gather the medication and supplies, including new tubing for secondary line, prepare the IV bag, and then exit the medication room through the same doors you entered. If other individuals are working at the Automated Dispensing Cabinet (ADC), please interrupt them to access you medication as quickly as possible. Once you have gathered meds and supplies, you exit the medication room through the same doors you entered (door 1).

To open the med room door, touch the card reader (black box beside the doors). Place your hands under the faucet, soap, or hand sanitizer to indicate usage. To retrieve the medication, touch the ADC computer screen which will open an ADC drawer. Injectable medications can be prepared by gathering the medications and supplies needed, placing them onto a work surface and holding the progress ball (symbolizes time typically used preparing the IV bag). Supplies are stored on the supply shelf and in the drawer below the med prep areas with the computers. When preparing ondansetron, assume the patient already has a primary line running in their room so you only need the secondary line.

Nurse 3 Tasks

Enter medication room from door 1 without a Wi-Med cart
Wash hands at the sink

Prepare Medication for Patient I

- interrupt person using ADC to urgently access medication from the ADC for Patient I
  - ondansetron inj 4 mg IVPB once, start at 08:00, requires secondary line only, (from ADC, in 50ML 0.9% NaCl)
- gather the following for the preparation of Ondansetron
  - medication added label, orange (from drawer below med prep area)
  - IV line label, white (from drawer below med prep area)
  - alcohol swabs (from drawer below med prep area)
  - blunt fill needle (from large supply shelves or drawer below med prep area)
  - 5 mL syringe (from large supply shelves)
  - 50 mL 0.9% Sodium Chloride injection USP (from large supply shelves)
  - secondary medication set (from small supply shelves)
- prepare IV bag
  - hold progress ball at work surface until task is completed
  - throw out garbage
  - put syringe and needle into sharps bin
  - put IV bag and line label into patient bin

Put secondary medication set onto Wi-Med cart
Wash hands at the sink
Exit door 1

Scenario: Pharmacy Technician 1

You are about to stock the med room with medications. To do this you will be taking your pharmacy cart into the medication room and placing medications from within the top drawer of your cart into its appropriate storage location. Once you have emptied the top drawer of your pharmacy cart, you exit the medication room through the same entrance (door 2) you entered.

To open the med room door, touch the card reader (black box beside the doors). Place your hands under the faucet, soap, or hand sanitizer to indicate usage. Once you open the top drawer of the pharmacy cart, you will see a label with the medication name and appropriate storage location. Pick up the medication, touch the Automated Dispensing Cabinet (ADC) to indicate that you are stocking the medication and then place the medication in the appropriate storage location. Re-open the top drawer until there are no remaining medications.

Pharmacy Technician 1 Tasks

Enter the medication room (1 minute after Nurse 2) from door 2 with a pharmacy cart
Use the hand sanitizer

Stock Medications

- stock medications from the pharmacy cart into the ADC, fridge and supply cabinet
    - acetaminophen tab 352 mg (into ADC [NOTE: involves picking up med, entering {touching} into ADC computer, drawer and bin lid auto opens, put into bin])
    - thiamine tab 100 mg (into ADC)
    - amoxicillin/clavulante tab 875 mg / 125mg (into ADC)
    - Ibuprofen tab 800 mg (into ADC)
    - metoPROLOL tab 50 mg (into ADC)
    - pantoprazole 40 mg (into ADC)
    - piperacillin / tazobactam inj 4.5 g IVPB, in 100ML D5W (enter in ADC, then directed to bin in fridge)
    - ampicillin 1 g, in 0.9% NaCl 100 mL Minibag Plus (enter in ADC, then directed to bin in fridge)
    - KCl 40 mmol in 0.9% NaCl infusion 1,000 mL IV (into large supply shelves)
    - salbutamol inhaler (into ADC)
    - diclofenac 2.32% gel (into ADC)
    - dalteparin inj 5,000 unit (into ADC)

Exit the room through door 2 with the pharmacy cart
